# Natural, Persistent Oscillations in a Spatial Multi-Strain Disease System with Application to Dengue

**DOI:** 10.1371/journal.pcbi.1003308

**Published:** 2013-10-24

**Authors:** José Lourenço, Mario Recker

**Affiliations:** Department of Zoology, University of Oxford, Oxford, United Kingdom; University of Michigan and Howard Hughes Med. Inst., United States of America

## Abstract

Many infectious diseases are not maintained in a state of equilibrium but exhibit significant fluctuations in prevalence over time. For pathogens that consist of multiple antigenic types or strains, such as influenza, malaria or dengue, these fluctuations often take on the form of regular or irregular epidemic outbreaks in addition to oscillatory prevalence levels of the constituent strains. To explain the observed temporal dynamics and structuring in pathogen populations, epidemiological multi-strain models have commonly evoked strong immune interactions between strains as the predominant driver. Here, with specific reference to dengue, we show how spatially explicit, multi-strain systems can exhibit all of the described epidemiological dynamics even in the absence of immune competition. Instead, amplification of natural stochastic differences in disease transmission, can give rise to persistent oscillations comprising semi-regular epidemic outbreaks and sequential dominance of dengue's four serotypes. Not only can this mechanism explain observed differences in serotype and disease distributions between neighbouring geographical areas, it also has important implications for inferring the nature and epidemiological consequences of immune mediated competition in multi-strain pathogen systems.

## Author Summary

The population dynamics of multi-strain pathogens are often characterized by persistent and irregular fluctuations in disease incidence and strain prevalence levels over time. Previous theoretical approaches have often evoked strong immunological interactions between individual strains, such as cross-immunity, in order to explain these complex epidemiologies; however, spatial segregation between hosts and stochastic heterogeneities in transmission success are rarely considered in these studies. Here, with specific reference to dengue, we show that the stochasticities underlying disease transmission within a spatially explicit, agent-based model can give rise to multi-annual epidemic outbreaks and fluctuating pathogen population structures - even in the absence of immune competition. In contrast to previous modeling studies, which have resulted in ambiguous predictions about the exact nature and strength of interactions between dengue's four serotypes, our results present a parsimonious, demographic mechanism, that highlights the importance of spatial ecology for understanding and interpreting the epidemiological dynamics of dengue and other multi-strain pathogen systems.

## Introduction

Mathematical models based upon the various derivatives of the classic susceptible-infected-recovered (*SIR*) framework have greatly improved our understanding of the transmission and population dynamics of many important pathogens [1], [2]. Common to this class of models is their propensity to exhibit damped oscillations around an approaching equilibrium where the rate of new infections equals the loss from the infectious pool due to recovery. In reality, however, many infectious diseases will not remain in this state of equilibrium but instead exhibit persistent oscillations, ranging from seasonal increases in incidence rates to multi-annual epidemic outbreaks. Measles and influenza are just two examples of pathogens for which incidence levels can vary by orders of magnitude within a single year [3], [4]. External forces are often incorporated into models to reflect a seasonal increase or decrease in the number of infectious contacts or vector densities, for example, which move the system's dynamics away from its natural equilibrium into a regime characterised by periodic or chaotic oscillations, akin to those observed in nature [5],[6].

For antigenically diverse pathogens, periods of high or low infection rates or the timing by which one dominant antigenic strain is replaced by another strain, are often out of sync with those dictated by the external forces, however. Theoretical studies have therefore concentrated on biological or pathogen-intrinsic factors instead. Immunological interactions between the constituent strains in the form of cross-immunity or cross-enhancement have been repeatedly highlighted as some of the most important determinants of the epidemiological dynamics of multi-strain pathogens. Under this scenario, enhanced competition for susceptible hosts can offer a temporary selective advantage to a particular strain or subset of strains, causing their amplification and subsequent decline. This process of immune-mediated selection has been proposed to underlie the population biology of a variety of important pathogens, including the influenza virus [7], *Plasmodium falciparum* [8], *Vibrio cholerae* [9], dengue virus [10]–[12], respiratory syncytial virus [13] and rotavirus [14].

Whereas many deterministic multi-strain models rely on the presence of immune interactions to destabilize the system, existing natural variabilities or stochasticities in the interactions between the relevant players have also been shown sufficient to generate regular or chaotic oscillations in single-strain and ecological predator-prey systems [15]–[19]. Furthermore, demographic stochasticities have been found to play an important role when relaxing the assumption of homogeneous mixing and when taking spatial ecological aspects into consideration. In this scenario, spatial heterogeneities due to host-population structure or local ecological variations can create short-lived spatial refuges [20] and significantly affect pathogen persistence [21]–[23].

The consideration of spatio-temporal variations is of particular importance for vector-borne pathogens, where the underlying drivers of the observed epidemiologies may be confounded by substantial heterogeneities in host and vector densities through space and time, as in the case of the dengue virus (DENV). DENV's population comprises four antigenically related viral groups, or serotypes (DENV1-4), that are the cause of clinically indistinguishable illnesses in humans. Different immunological interactions in the form of antibody-dependent enhancement (ADE) or temporary and/or partial cross-immunity have been independently proposed as the driving forces behind the virus' complex epidemiology that comprises multi-annual epidemic outbreaks and sequential replacement of dominant serotypes [10]–[12], [24]–[27]. Although these differential equation models qualitatively capture dengue's epidemiological dynamics, they do not consider the natural variability in disease transmission across time and space and thus cannot account for observed differences in incidence and serotype distribution within endemic regions (see e.g. [28]). Meta-population and agent-based models allow a more explicit description and investigation of demographic and spatial, ecological stochasticities [29]–[31], and thus provide a natural alternative to study these host-pathogen systems.

Here, using dengue as a case study, we show that heterogeneities and stochasticities underlying host-vector contacts can give rise to persistent oscillations in multi-strain pathogen systems, even in the absence of immune competition between antigenic types. We demonstrate that viral persistence is significantly enhanced through the temporal generation of susceptibility pockets within the population, leading to highly heterogeneous distributions in disease and serotype prevalence that can explain observed geographic differences in dengue endemic regions. Complimentary to immune interaction, host demographic factors and vector ecologies thus emerge as important drivers of dengue's epidemiological dynamics.

## Results

To examine the effects of spatial structuring and stochasticities in disease transmission on the qualitative dynamics of a multi-strain pathogen system, and understand how this might help explain some of the observed epidemiological features of dengue, we first extended a previously analysed single-strain model with homogeneous mixing [18] to incorporate multiple strains; for this part we used the original parameter set for direct comparison between this extended and the original model (see Materials and Methods). We then developed this model into a dengue-specific framework by including mosquito vectors, seasonality and spatial population structuring, together with dengue-relevant parameter values (see Table 1). Unless stated otherwise, we analysed the dynamical behaviour of these models in the absence of immunological strain interactions except for the prevention of superinfection. The epidemiological frameworks of both models are detailed in the Materials and Methods section.

**Table 1:** Parameters and values for 4-serotype dengue model.

Parameter	Description	Value [range]	Reference
1/*δ_h_*	intrinsic incubation period	2 days [2–7]	[66]
1/*γ_h_*	human infectious period	4 days [4–12]	[67], [68]
1/*α_h_*	period of temporary cross-immunity	2–12 months [2–12]	[46]
* 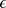 _hν_*	human-to-vector transmission probability	0.5 per bite [0.33–1]	[46], [69]
1/*ν_h_*	average life-span (human)	60 years	- -
*φ_h_*	enhancement in secondary heterologous infections)	1–2	- -
*M*	vectors per human host	0.7–1.2 [0.3–20]	[70]
*a_v_*	mosquito biting rate	0.6 per day [0.33–1]	[71]
1/*δ_v_*	extrinsic incubation period	6 days [6–12]	[51]
* 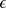 _vh_*	vector-to-human transmission probability)	0.5 per bite [0.33–1]	[46], [69]
1/*ν_v_*	average life-span (mosquito)	23 days [8–42]	[71], [72]
*μ*	external infection rate	0.000005 per day	- -
*N_h_*	human host population size	100000	- -
*ω*	human daily mobility	[0–1]	- -
*a_h_*	Weibull scale (humans)	0.0055	- -
*b_h_*	Weibull shape (humans)	5.5	- -
*a_ν_*	Weibull scale (mosquito)	0.04	- -
*b_ν_*	Weibull shape (mosquito)	4	- -

### Non-spatial dynamics

**Figure 1:**
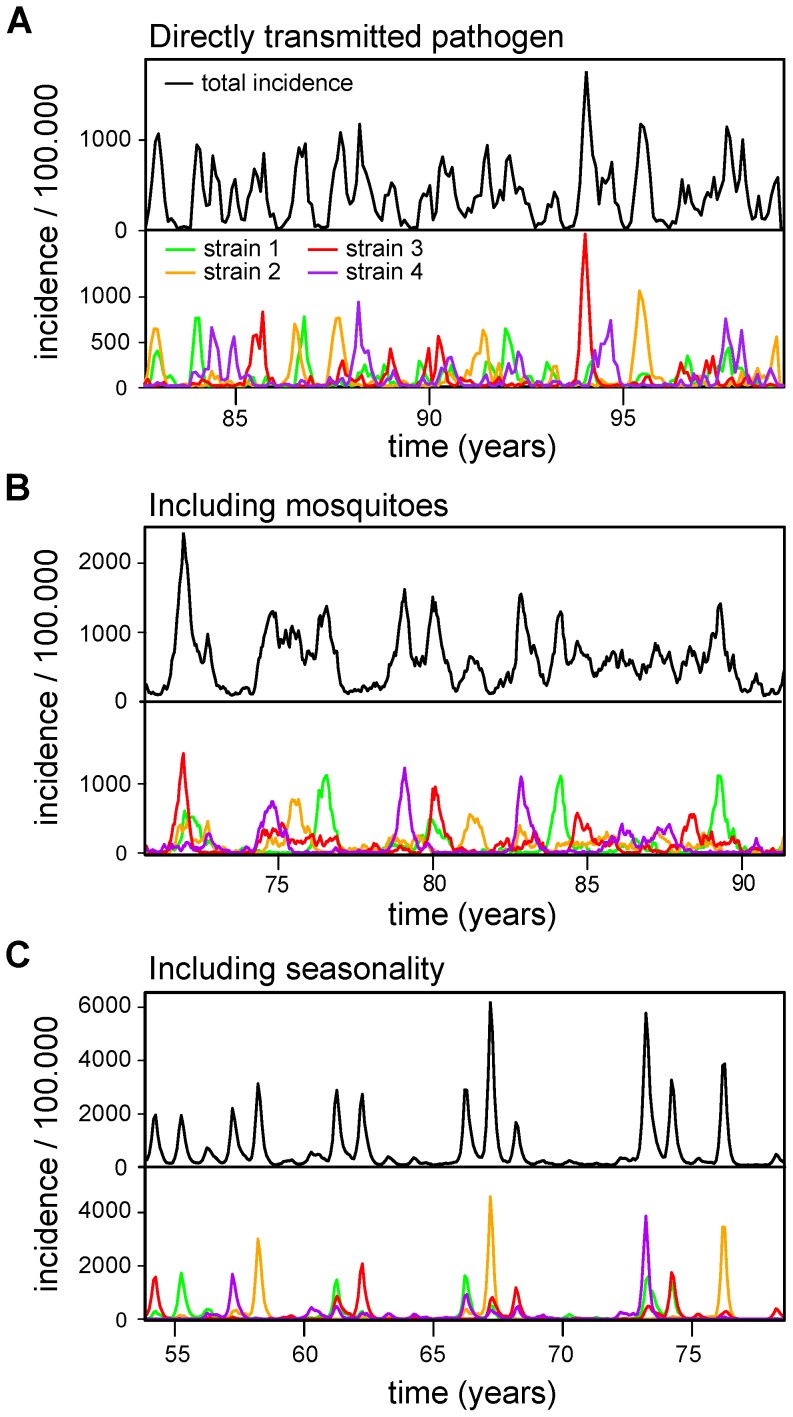
Epidemiological dynamics of a multi-strain system with homogeneous mixing. (***A***) The simulated time series of an agent-based, multi-strain model of a directly transmitted pathogen show irregular oscillations in total (black lines, top graphs) and strain-specific (coloured lines, bottom graphs) even in the absence of immunological interactions or asymmetries between pathogen strains. (***B***) Changing the system to describe a vector-transmitted pathogen, including intrinsic and extrinsic incubation periods, results in an overall increase in mean incidence and decrease in the risk of stochastic extinction. (*C*) Further including seasonal variations in mosquito densities results in multi-annual epidemic outbreaks followed by severe transmission bottlenecks. Parameters are given in Materials and Methods (A) and in Table 1 (*B* and *C*, with 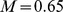 in *B*).

Using a stochastic, agent-based framework we first analysed the dynamics of a host-pathogen system comprising 4 co-circulating antigenic types under the assumption of homogeneous mixing within the population. Contrasting the predictions of deterministic multi-strain models, in which the dynamics inevitably converge towards a stable equilibrium in the absence of strong immune competition, the system exhibited sustained oscillations in the total number of infections and out-of-phase oscillations in strain prevalence, as illustrated in Figure 1A. In agreement with previously studied stochastic single-strain systems [17], [18], these dynamics are driven by the amplification of stochastic effects at the individual level, which keep each strain in a transient regime rather than approaching the expected deterministic equilibrium. At the same time, short-term stochastic differences in each strain's transmission success accumulate in time and start to generate significant asymmetries in the immunity profile within the host population, which then leads to the desynchronisation between strains.

This extreme case of minimal strain interaction more resembles a system of four co-circulating but unrelated pathogens. Not surprisingly, therefore, we found that the periods of oscillations in total incidence and strain prevalence were essentially the same, determined by the parameters relating to pathogen transmission and host demography (Figure S1A in electronic supplementary material). In the case of dengue, however, differences between the inter-epidemic period and average cycle length in strain prevalence have been well documented [32]. We therefore extended the model to incorporate mosquito vectors and used dengue-relevant epidemiological parameters values (see Table 1) to investigate the effect of stochastic amplifications on the virus's epidemiological dynamics and inter-epidemic periods. The resulting qualitative dynamics in terms of persistent oscillations in incidence and serotype prevalence appeared invariant to the addition of mosquito vectors but showed a significant increase in average disease prevalence (Figure 1B). This increase was mainly caused by a reduction in the risk of stochastic extinction due to the inclusion of viral incubation periods as well as the increase in the basic reproductive number from 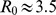 in the directly transmission model to 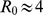 in the vector model. Importantly, also, we started to observe a divergence between the epidemic and serotype periodicities (figure S1B in electronic supplementary material) and also found epidemic peaks more likely to be comprised of multiple serotypes.

Further including seasonality through annual variations in mosquito densities (see Materials and Methods) resulted in dengue-like epidemiological behaviour with a distinct seasonal signature, strong multi-annual periodicities in incidence and fluctuating distribution in serotype prevalence (Figure 1C). This behaviour was further accompanied by a considerable increase in peak incidence levels and more pronounced epidemic troughs, which could partly be explained by an increase in the average 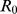 to 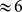 but also by the strong synchronizing effect of vector seasonality on serotype dynamics.

### Spatial dynamics of dengue-specific model

We hypothesized that the occurrence of large epidemic outbreaks (as seen in Figure 1C) was partly facilitated by our assumption of homogeneous mixing, which facilitates rapid disease transmission throughout the whole population. We thus restructured our model into a meta-population formulation by subdividing the human and mosquito populations into sets of spatially arranged communities (see Materials and Methods) and examined the effect of spatial segregation between hosts on the epidemiological dynamics of this multi-strain system. Within this set-up we assumed that individuals get infected predominantly by mosquitoes of their own and surrounding communities and with a small probability, 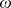, by mosquitoes from distant communities through (temporal) human movements, or visits, to these communities. We argued that because of the limited flight range of *Aedes* mosquitoes, human movement is more important for long-distance transmission [33] and therefore assumed 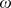 to be independent of geographic distance, contrasting continuous and distance-dependent dispersal kernels often employed in spatial ecological models (but also see [33] and [34] for alternative realisations).

**Figure 2:**
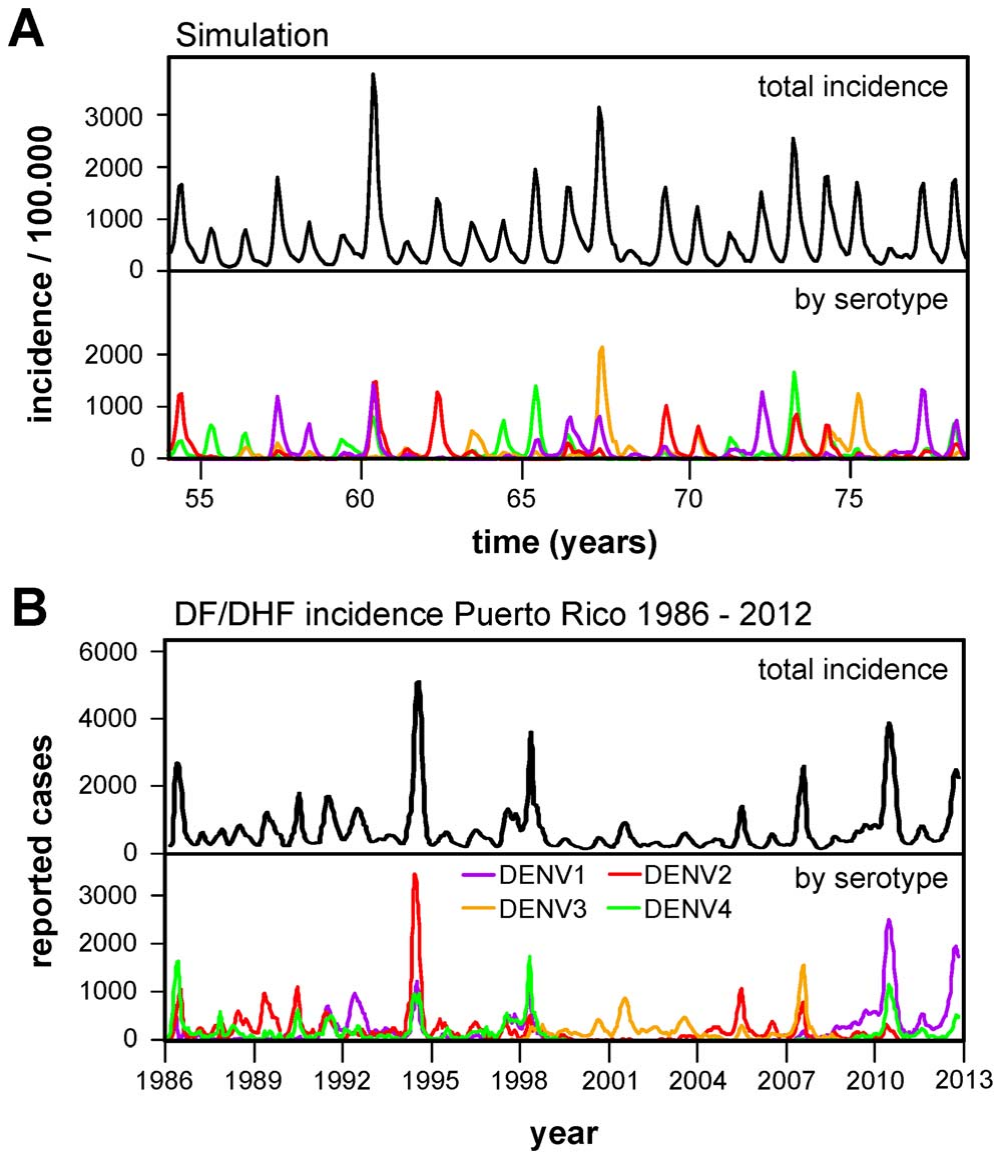
Temporal epidemiological patterns of dengue. (***A***) Model output. Structuring the host population into a (20 by 20) lattice of smaller sub-communities results in lower epidemic variability in the simulated epidemiological dynamics and higher out-of-season viral persistence. The average level of disease prevalence is 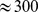 per 100000 individuals and the proportion of the population fully susceptible to dengue is 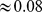. Parameters as in Table 1 with 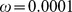. The overall qualitative behaviour in incidence and serotype oscillations are in good agreement with dengue characteristic epidemiologies. (***B***) Empirical data. Time series of reported cases of DF and DHF in Puerto Rico in the period 1986–2012 (top) showing a clear seasonal signature and multi-annual epidemic outbreaks. Plotting adjusted serotype-specific incidence (bottom) illustrates the sequential replacement of dominant serotypes over time.

With the addition of this spatial component the system exhibited more defined seasonal dynamics as well as a lower variability in the epidemic behaviour (Figure 2A), with the overall temporal dynamics closely resembling epidemiological time series from dengue endemic regions with the characteristic multi-annual cycles in epidemic outbreaks and sequential serotype dominance (Figure 2B showing data from Puerto Rico, and Figure S2 showing data from Thailand, Mexico and Vietnam). The periodicity in serotype prevalence also increased and settled onto a 8–9 year cycle (figure S1C in electronic supplementary material), which is in line with the suggested periodicity derived from epidemiological time series [32] (also apparent from Figure 2B) and dengue's phylodynamics in Thailand [25].

**Figure 3:**
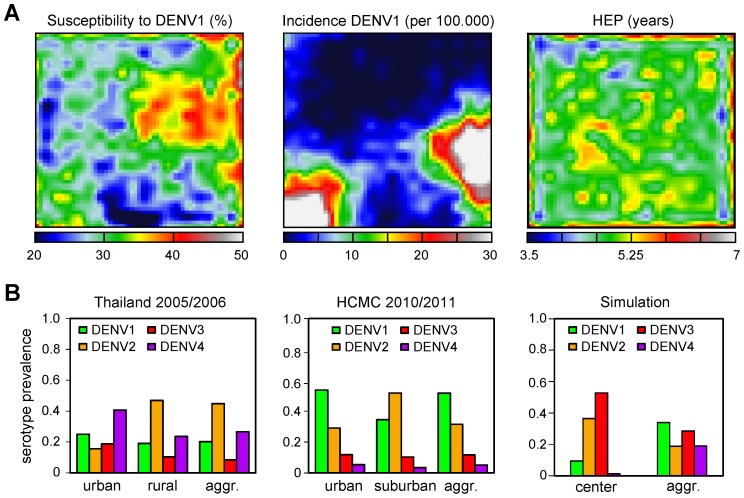
Spatial epidemiological patterns. (***A***) Local viral extinction generates a highly heterogeneous immunity landscape, shown as a snapshot (at year = 80) of the population-wide susceptibility level to DENV1 (*left*). The spatial prevalence of individual serotypes is equally heterogeneous, driven by serotype-specific susceptibility and here shown as the cumulative incidence of DENV1 for the following 3 seasons (*middle*). Spatial heterogeneity in serotype prevalence and exposure causes a highly variable distribution in the heterologous exposure period (HEP), or timing between consecutive, heterologous infections(*right*). (***B***) Significant differences in serotype prevalence can be observed on multiple geographical scales during a single season within endemic regions, which would be hidden by just considering aggregated data: between rural and urban Thailand (*left*) and within Ho Chi Minh City (*middle*). Simulation output (*right*) showing similar patterns in serotype distribution, where a community in the center of the lattice exhibits dissimilar serotype prevalence levels compared to the aggregated meta-population data, taken from the last 2 years of the simulation shown in Figure 2A.

In agreement with previous studies on meta-populations, the spatial segregation between hosts enhanced global disease persistence (compare e.g. baseline incidence in Figures 1C and 2A) but at the same time facilitated local extinction [17], [22], [35]. This created a spatially heterogeneous susceptibility landscape within the population (Figure 3A, left panel) upon which individual serotypes were sequentially selected and amplified, frequently exhibiting locally propagating waves (Figure 3A, middle panel). This heterogeneity in susceptibility and disease prevalence also affected the timing between heterologous infections, here referred to as heterologous exposure period, or HEP, leading to a highly variable, spatio-temporal distribution in HEP across the population (Figures 3A, right panel). We argued that these self-emergent phenomena could explain some of the spatial epidemiological differences in dengue-endemic countries, where markedly different distributions in serotype prevalence can be observed between geographically neighbouring regions or between urban and suburban districts (Figure 3B). Importantly, these differences would be masked if only aggregate data were being considered.

### The effects of population structure and host mobility

The spatio-temporal dynamics illustrated in Figures 2 and 3 clearly demonstrate the importance of human and vector demographic heterogeneities for the population dynamics of dengue [36]–[38], which in our case are the result of stochasticities and spatial restrictions in disease transmission. To further address the effects of spatial structuring and host mobility on our simulated epidemiologies, we quantified key epidemiological properties, such as mean prevalence (averaged over humans and vectors), extinction risk and serological age-profiles in the population, in response to changes in these parameters.

**Figure 4:**
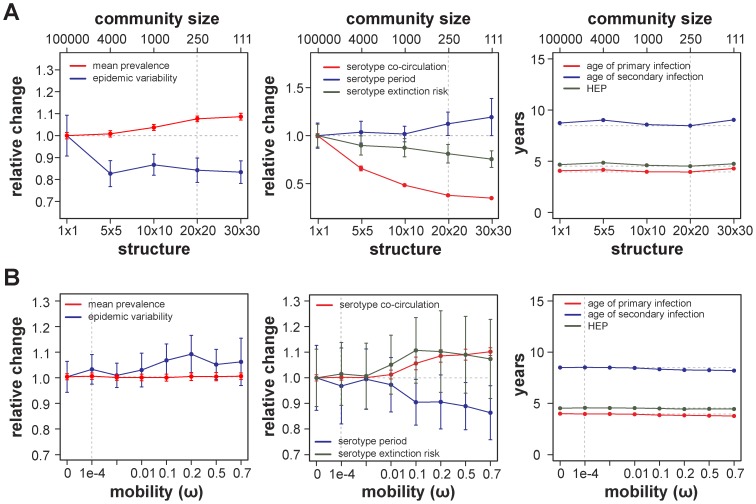
Effects of population structuring and host mobility. (***A***) Increasing host population structure results in a significant reduction in epidemic variability (blue line in left panel), extinction risk (green line, middle panel), longer periods of serotype oscillations (blue line, middle panel) and serotype co-circulation (red line, middle panel). This increase in viral persistence also causes higher mean prevalence (red line, left panel). The age of primary or subsequent infections are not affected by changes to population structuring (right panel). (***B***) Host mobility, 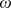, counteracts the effects of population structure (here using a 20×20 lattice) and leads to an increase in epidemic variability and therefore extinction risk. In both (*A*) and (*B*), the average age of infection is not affected as the mean force of infection is maintained. Note, the oscillatory behavior in serotype prevalence is maintained given the parameter variations, with periods between 7 and 10 years in (*A*) and between 7 and 9 years in (*B*). Extinction risk is defined as the percent of time individual serotypes remain bellow a critical threshold of 10 infected hosts (human or mosquito). For ease of comparison, epidemiological variables (except age) are normalised to the case of no structuring in (*A*) and no host mobility in (*B*), with ratios above 1 representing an increase and below 1 a decrease. Dashed vertical and horizontal lines mark the parameter set of Figure 2. Shown are the means and deviations for 25 stochastic simulations.

Increasing spatial structuring, and thereby decreasing the size of each sub-population, reduced the variability in total annual outbreak size and local serotype co-circulation (Figure 4A), here defined as the percent time where multiple serotypes are present in a given patch. Although the overall force of infection was not affected by the increase in population structure, as evidenced by the constant average ages of primary or secondary infections (right panel, Figure 4A), total infection prevalence increased as a result of a reduction in the risk of serotype extinction. This indicates that spatial segregation between hosts greatly reduces the propensity for large-scale, population-encompassing outbreaks by restricting a pathogen's access to the susceptible pool, which is also in agreement with previous studies in the context of disease transmission through complex or/and heterogeneous networks [17], [35], [39].

In contrast to population structuring, increasing the probability of transmission between hosts of distant communities, 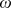, as a proxy for daily human mobility, had a more homogenizing effect and led to an increase in local viral co-circulation (Figure 4B). More frequent and brief localized outbreaks could be observed, resulting in increased epidemic variability. However, this increase in outbreak size variability did not equate to an increase in mean infection prevalence levels because of localised extinction risk. In other words, the heterogeneous distribution of herd-immunity [40] to individual serotypes (as illustrated in Figures 3A–C) within the spatially structured population counteracts the occurrence of population-wide outbreaks that are otherwise expected from the synchronizing effect of higher mobility or dispersal rates [22], [41].

### The effect of host mobility on spatial synchrony

**Figure 5:**
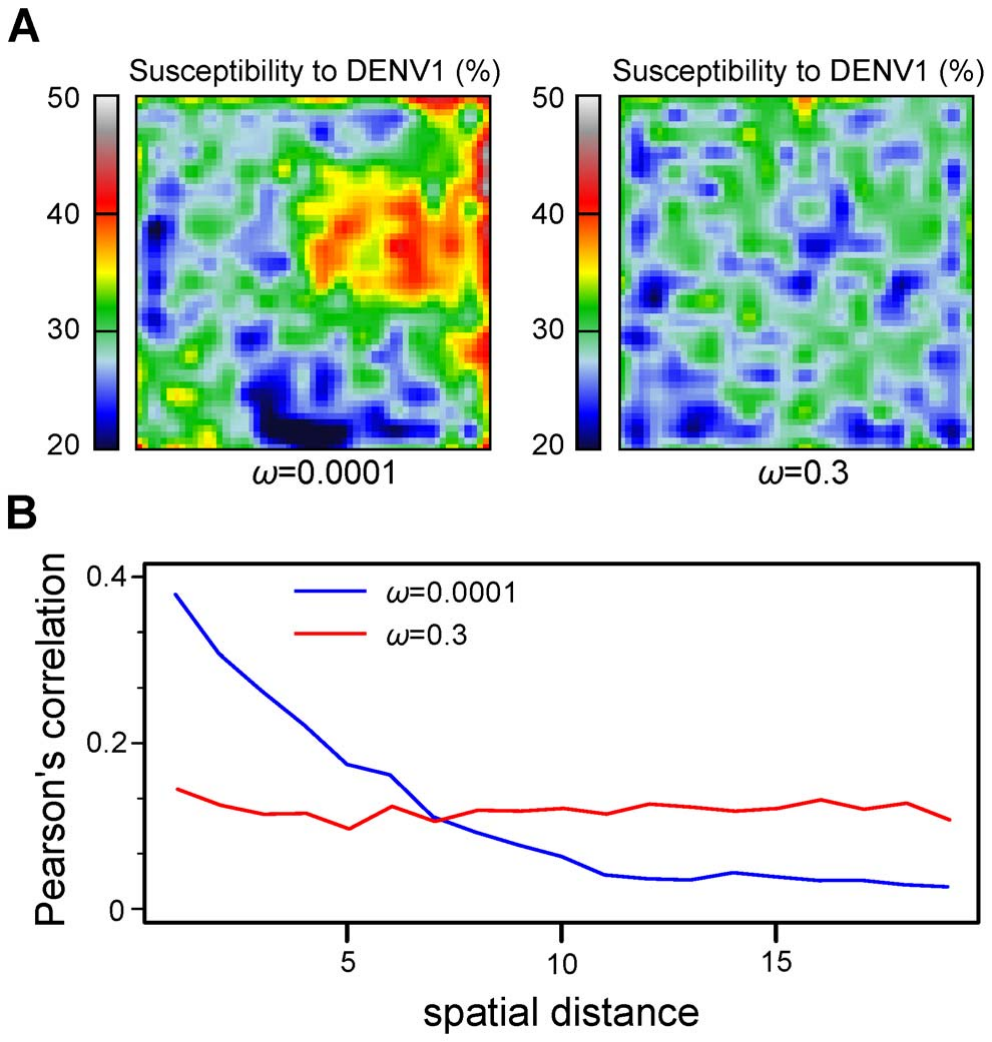
Effects of host mobility on spatial coherence. (***A***) Increasing the probability of long-distance transmission, 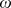, as a proxy for increased daily (human) mobility, results in a less variable but more patchy immunity landscape across the population, as shown as a snapshot of the DENV1 susceptibility levels across the population. (***B***) This effect on spatial heterogeneity in population-level immunity is also reflected in terms of spatial coherence between communities, here shown as Pearson's *r* between communities along the diagonal. Whereas predominantly local transmission results in a sharp decrease in spatial coherence with distance (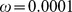, blue line), high host mobility leads to a generally low and homogeneous degree of coherence across the population (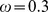, red line), due to the nature of mobility here assumed to be stochastic both in time and space.

We next analysed the degree of epidemic synchrony, or coherence, between communities under variations in host mobility. As mentioned above, the rate at which human hosts acquire infections in geographically distant communities, 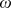, has a significant effect on viral co-circulation and hence the susceptibility/immunity landscape in the population. This is further illustrated in Figure 5A for two different values of 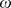, showing a transition to a less variable but a more patchy distribution of susceptibility to DENV1 with an increasing rate of long-distance transmission events. When disease transmission was predominantly local (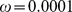), as expected, we observed that spatial synchrony was dependent on spatial distance (blue line in Figure 5B, and Figure S3). In contrast, as a result of a reduction in locally acquired infection with increasing 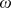, epidemic synchrony between neighbouring communities was disrupted, causing an overall low but homogeneous spatial coherence across the population (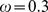, red line in Figure 5B). These results are in general agreement with a growing body of studies on dengue's epidemic, spatial scale. For instance, cases appear to cluster at the level of households or neighborhoods [42], whereas epidemics across larger regions present strong spatial dependence [43] and appear to follow a power-law distribution, implying that outbreaks are predominantly driven by a limited set of spatial clusters [44].

It should be noted that migration, or dispersal, has previously been shown to increase synchronization between populations within different spatially explicit model frameworks [22], [41]. However, this is not necessarily the case when local demographic stochasticity is considered [19], [34]. For instance, within a spatially extended meta-population model, Blasius *et al.* demonstrated that phase-locking amongst patches is easily achieved by dispersal rates, while peak and trough abundances in each patch can remain chaotic and variably uncorrelated [34]. The same effect is observed in the local dynamics of the patches within our framework (see Figure S3A and S3B for examples). It is thus not surprising that we only find low-to-intermediate coherence across space, even between close-range patches (Figure 5B).

### The effects of serotype immune interactions

**Figure 6:**
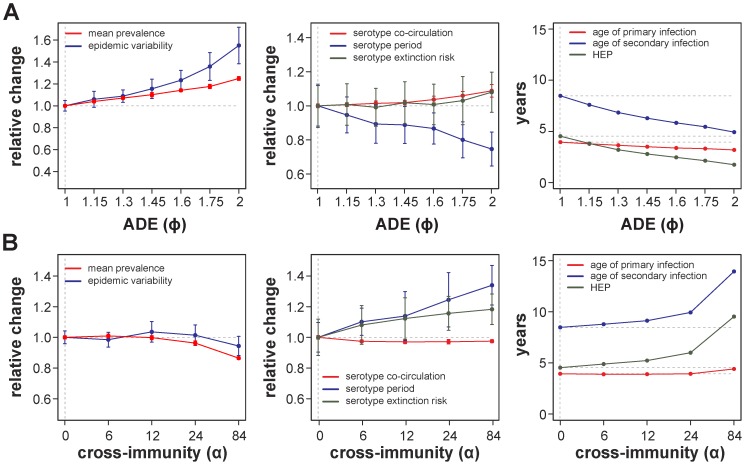
Effects of serotype immune interactions within structured populations. (***A***) The epidemiological effects of temporary cross-immunity, 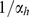, on mean prevalence level, epidemic variability or average age of infection only become apparent when the period of immunity increases beyond 12–24 months. Longer periods hamper variant transmission and lead to a decrease in mean disease prevalence and significant increase in the age of heterologous infection. (***B***) Antibody-dependent enhancement, 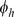, which simultaneously increases susceptibility to and transmissibility of secondary, heterologous infections causes an overall increase in the force of infection and more variable epidemic behaviour. Due to higher susceptibility and co-circulation this also leads to a drop in the age of primary and particularly secondary infection. The oscillatory behavior in serotype prevalence is maintained given the parameter variations, with periods between 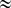
8 and 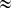
12 years in (*A*) and between 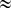
6 and 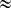
9 years in (*B*). For ease of comparison, epidemiological variables (except age) are normalised to the case of no cross-immunity, with ratios above 1 representing an increase and below 1 a decrease. Dashed vertical and horizontal lines mark the parameter set of Figure 2. Shown are the means and deviations for 25 stochastic simulations.

Although dengue-characteristic dynamics could be obtained even in the absence of immune interaction between the virus's four serotypes, temporary (serotype-transcending) cross-immunity and ADE have previously been proposed as important drivers of dengue epidemiology, and we therefore analysed their effects within this spatial setting. As demonstrated in Figures 3A and 4 (right panels), the time required for an individual to acquire a secondary, heterologous infection (HEP) was on average in the order of 4–5 years. While this is in general agreement with a previous study from Thailand [45], and might also explain the peak in older children in the age-profiles of dengue haemorrhagic fever (DHF) in endemic regions [32], it is much higher than the reported 3–9 months period of serotype-transcending immunity following a primary infection [46]. Consequently, and contrary to previous predictions based on continuous and homogeneous mixing models, the inclusion of temporary cross-immunity did not have a significant effect on the simulated, qualitative epidemiologies within our stochastic and spatially explicit framework. When quantifying key epidemiological characteristics under changes to the duration of temporary immunity, we found that only once this period increased beyond 12 months there was a small, negative effect on infection prevalence and epidemic variability (Figure 6A). On the other hand, even short periods of transcending immunity had a significant effect on both serotype extinction risk and periodicity, suggesting its regulatory role on how the different viruses can explore the susceptibility (spatial) landscapes.

In contrast to temporary cross-immunity, immune enhancement through the process of ADE had a more noticeable and anticipatory effect. That is, increasing the probability of transmission through the enhancement of secondary, heterologous infections led to an increase in disease prevalence along with an increase in epidemic variability, serotype co-circulation and viral extinction risk (Figure 6B), which is broadly in line with previous studies [10], [12], [24]. The increase in prevalence did not significantly affect the average age at which individuals experience their first infection, however, whereas the age of secondary infection showed a more dramatic reduction. In fact, due to the combined effect of elevated serotype co-circulation and an increase in the susceptibility to secondary infections through ADE, even moderate levels of enhancement caused the HEP to go below the average time at which individuals experience their first infection. Hence, in the presence of population structure, ADE, and especially its proposed susceptibility enhancing manifestation, may induce a signature in the epidemiological age-profiles of the population that is characterised by a longer period for first infection than the time required for heterologous exposure, which has indeed been observed in studies of clinical infections in dengue endemic areas [32], [45], [47].

### Model behaviour under changes in 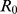


Finally we turned our attention to the effect of changes to disease transmission within this spatial setting. Differences in the estimates of a pathogen's transmission potential, or 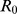, can be attributed to a multitude of factors, and in the case of dengue, this has resulted in a wide spectrum of estimations, ranging in values from close to 1 to bigger than 20 (see Table S1 for an overview). To quantify the effects of changes to viral transmission, and 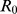 in general, we analysed the model behaviour, in the absence of immune interactions, under variations in key parameters related to dengue's basic reproductive number, whose derivation within this framework can be found in the Materials and Methods section.

Specifically, we investigated the effects of 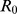 through variations in the probability of transmission per mosquito bite, using both symmetric and asymmetric transmission probabilities, viral incubation periods (both intrinsic and extrinsic) and mosquito vector density. The results were mostly in accordance with those expected from increasing parameters related to 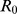 in equivalent continuous multi-strain models and can be found in Figure S4 in Supplementary Material; here, we only highlight two of the more important findings. First, assuming symmetric transmission probabilities between host and vectors we found that the viral extinction risk was not monotonously associated with changes in transmissibility and was in fact minimized for 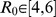 (Figure S4A), in range with estimations using age-stratified indexation of sero-conversion rates [48]. Notably, this was also the range in which the age profiles of infection where more similar to what is commonly described for endemic regions in South East Asia [32], [45], [49], [50]. Secondly, our model confirmed that changes in the extrinsic incubation period had a much more dramatic effect on dengue epidemiology than incubation periods in the human host (Figure S4D and S4E), as this directly affects the duration of infectiousness in the mosquito. Crucially, this re-emphasizes the notion that seasonally driven temperature, and its effect on viral incubation, is as important a determinant of dengue epidemiology, as is vector density [51].

## Discussion

Understanding the evolutionary forces that shape the spatio-temporal patterns of pathogen populations is essential for disease control and public health planning. Important new insights into the population dynamics of host-pathogen systems have been gained by the application of deterministic mathematical models to the study of many important infectious diseases [1]. Nevertheless, stochastic and discrete events significantly influence the real world counterpart of such systems and their explicit incorporation can provide alternative frameworks in which to examine major determinants of the observed epidemiologies [17], [39], [52], [53]. In this context, demographic stochasticity has been suggested to be an important driver for population oscillations in single-strain epidemiological systems [5], [6], [17], [18]. Here we advanced upon previous findings by studying the dynamical behavior of dengue's four antigenic types within a stochastic and spatially explicit framework.

Dengue's epidemiological dynamics have been the focus of extensive theoretical research that often concentrated on the immunological interactions between its four serotypes [10]–[12], [24]–[26]. Protective and infection-enhancing effects of cross-reacting antibodies have been well documented both *in vivo* and *in vitro* [46], [54], [55]. Less clear, however, is their contributing effect to disease transmission and general epidemiology. For example, although a short period of 3 to 9 months of serotype-transcending immunity following a primary infection has been demonstrated by direct experiment, the average time between consecutive, heterologous infections is often found to be an order of magnitude higher [45]. Equally, despite the reported increase in within-host viral replication through antibody-dependent enhancement of secondary, heterologous infections and observed correlations between disease severity and previous exposure, it is currently not known if and how much this increase in viral load contributes to total dengue transmission, especially when taking into consideration that severe, clinical cases may constitute only a small fraction of all dengue infections [32], [45], and that viraemia appears to peak earlier but also clears faster during secondary, heterologous infections [56].

In contrast to previous model predictions, our results could not ascertain a decisive role of either temporary cross-immunity or ADE in driving the complex epidemiological dynamics of dengue. That is, while our findings do not question the pathological or clinical significance of immune interactions *per se*, they suggest that the strength of within-host serotype interactions, and therefore the consequences of acquired immunity, are unlikely to be the sole drivers of the complex epidemiological dynamics of dengue. Crucially, the results herein presented further suggest that such cross-immunological reactions, at least within biological reasonable ranges, would not cause significant spatio-temporal signatures that could allow the inference of their presence to be unambiguously resolved from studying epidemiological time series alone. More detailed data, for example from human infectivity studies that relate infection history with clinical outcome and infection/transmission probabilities, are essential to close the gap in our understanding of the full transmission potential of dengue. Furthermore, to better understand the importance of host demographic factors and spatial ecology highlighted in this work, a phylodynamics approach could be considered in which the spatio-temporal evolution of dengue genotypes is simulated and compared to available data from different settings across the endemicity spectrum.

Dengue's recent molecular evolution is characterized by strong intra-serotype purifying selection with no clear trend for continuous antigenic change. As DENV has evolved to replicate efficiently in both the vertebrate and arthropod hosts, it is thought to express a compromise genome in which most structural mutations are expected to be deleterious and selectively removed from the population [57]. On the other hand, strong ecological bottlenecks and inter-serotype competition can severely hamper the emergence of viral mutants even if they express advantageous phenotypes [58]. The cyclical replacement of dengue's four serotypes is therefore not expected to be driven by the same inter-strain selective forces that have (reportedly) shaped the phylodynamics of antigenically rapidly evolving pathogens, such as influenza A [59], for example. It instead argues for a critical role of demographic and ecological stochasticities underlying both dengue's epidemiology and molecular evolution.

The strong impact of host population structures and mobility highlighted in this work also corroborates the hypothesis that DENV's (re-)emergence and world-wide success is mainly due to current demographic and ecological trends rather than viral adaptation [39], [57]. To understand dengue's epidemiology in the long-term, it is therefore crucial to establish how these meta-population disease dynamics correlate with evolutionary constraints and respective selective signatures. Importantly, the discrete nature of our framework and its meta-population formulation readily allow to explore more realistic population structures, including heterogeneities in (host and vector) population sizes and/or connectivity between sub-populations, for example by means of complex network structures, and to simulate viral evolution in time and space within these frameworks. Our model thus presents itself as a good starting point for a more thorough investigation of DENV's phylodynamics [60].

Accounting for community-specific vector control and drug intervention policies is equally possible within this meta-population formulation and constitutes another important extension for future studies on the control of vector-borne diseases. For example, candidate vaccines against dengue that are in advanced stages of clinical trials might require a prime-boost protocol running over a period of up to 12 months, which has been indicated as a potential concern due to the risk of severe disease during the time when antibody-levels are at sub-neutralizing titers [61], [62]. By reproducing the spatial heterogeneity in disease prevalence and serotype distribution we found the timing between consecutive, heterologous infections to be highly variable in space. Our observations thus reassert that spatially explicit epidemiological frameworks, as the one presented here, are essential for assessing the risks and efficacies of vaccine introduction strategies against dengue [62].

In summary, the results presented here have highlighted the importance of considering spatial segregation between individual hosts and vectors and stochasticities in disease transmission for understanding the epidemiology of dengue and other related pathogens. Previous theoretical studies have demonstrated that immune interactions can significantly influence the population dynamics of multi-strain pathogen systems. The inclusion of host and vector ecologies adds to this understanding and provides complimentary hypotheses about the underlying causes for the oscillatory nature in incidence and serotype distributions that commonly characterize their complex epidemiologies.

## Materials and Methods

### Individual-based models

To study the stochastic dynamics of a multi-strain pathogen we used an individual-based model, realised as a discrete-time, random process with finite state-space (Markov chain), is which a state refers to the host's epidemiological profile, such as infection status and immune history. Demographic, biological and ecological stochasticities were derived from the probabilistic nature of state transitions, e.g. in the probability that the bite of an infectious mosquito leads to an infection. The size of the host population was kept constant with deaths being replaced by newborns. We assumed an age-dependent risk of mortality for both humans and mosquitoes, described by the continuous Weibull distribution:
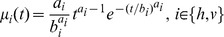
where 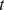 is the host age, and 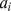 and 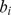 are the shape and scale parameters, respectively.

#### Direct transmission model

The initial, direct transmission model without spatial structuring (homogeneous mixing model) is based on the model analysed by Alonso *et al.* [18] but here extended to incorporate four co-circulating strains. We assumed contacts to take place between two individuals chosen randomly from the host population, with 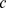 as the average number of contacts per individual per time step and 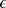 the probability of transmission from an infected to a susceptible host. For each pathogen strain (or serotype) 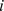, the expected number of infective contacts per time step was therefore given as 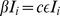. Following infection, individuals retained full immunity against the infecting strain but remained susceptible to all other strains; for simplicity we did not consider co-infection with two or more strains. For this model only, we used parameter values based on those from Alonso *et al.* [18]: transmission probability 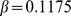, daily contact rate per host 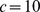, infectious period 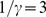 days, average host life span 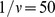 years, daily external infection rate 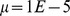, and host population size 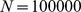. The basic reproductive number, 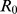, for this model is simply given as 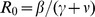, as in a classical SIR epidemiological model.

#### Dengue model

To model the population dynamics of dengue and its four serotypes (DENV1-4) we extended the initial model (see above) and added mosquito vectors to the system together with the virus's intrinsic (human host) and extrinsic (mosquito host) incubation periods, 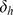 and 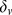, respectively. Only the susceptible, exposed and infectious states of the epidemiologically relevant adult life-stage of the mosquito were considered. To account for seasonal variation in vector densities, we assumed an annually driven mosquito birth rate, with the maximum number of vectors per human determined by 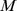, as previously used in [58]. Mosquitoes had a per-day biting rate of 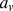 and, throughout the study, we assumed equal transmission probabilities in human-to-vector and vector-to-human transmission, 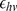 and 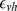, respectively (the effects of asymmetries in transmission probabilities can be found in supplementary material). To study the effect of immunological interactions between serotypes, we considered (i) temporary, serotype-transcending immunity, where individuals are fully protected against further challenge for a period 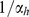 months after recovery, and (ii) antibody-dependent enhancement through an increase in susceptibility and infectivity of heterologous, secondary infections, 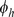. However, in the majority of our analyses, we did not consider immune interactions beyond the prevention of super-infections. For both the human and mosquito hosts we assumed age-dependent mortality rates (see above) with average life expectancies of 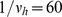 years and 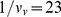 days for humans and mosquitoes, respectively. With the consideration of the mosquito-vector, the expression for the basic reproductive number changed to
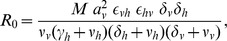
which has also been used by Wearing and Rohani [11]. We assumed an 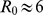, which is within the range of values estimated from dengue endemic settings and those used in previous theoretical studies (see Table S1 for an overview of 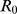 estimates, methodologies and references). Note, parameters values for this model were dengue-specific and therefore differed from those of the initial, direct transmission model. A full parameter list with values used, biological ranges and references can be found in Table 1.

### Host population structure

Spatial structure was added by subdividing the host population into a spatially organized set of communities, forming a squared and non-wrapping lattice wherein each community 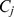 had 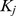 neighbors (). Individuals were assumed to mix homogeneously within each 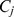, such that each mosquito bite took place between a vector and human chosen randomly from this community. We further assumed that mosquitoes disperse only locally, implying that each vector in community 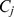 will only bite human individuals belonging to the set of communities 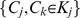, i.e. within 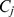 and its neighboring communities. Long distance transmission was considered through human movement by allowing mosquitoes to bite humans of randomly chosen, distant patches with probability 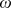 (the probability of human hosts temporarily ‘visiting’ these communities), which reduces the local transmission rate to 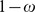. This formulation differs from the ones considered in other meta-population studies, which often assume a constant (continuous), and possibly distance-dependent migration or dispersal term between any two patches or communities.

### Dengue data

#### Temporal

Dengue incidence data for Puerto Rico (Figure 2) was provided by M. Johansson from the CDC Dengue Branch, comprising clinically suspected cases of dengue fever (DF) and dengue hemorrhagic fever (DHF) in Puerto Rico between 1986–2012. Serotype-specific testing has varied over time, so we adjusted the serotype-specific incidence data proportionally to match the incidence of all suspected cases by month. A slightly shorter time series had previously been published and analysed in [63].

Monthly incidence of suspected DF or DHF cases in Mexico 1985–2011 (Figure S2A, top) was obtained from the Mexican Secretariat of Health (www.epidemiologia.salud.gob.mx/dgae/infoepid/inicio_anuarios.html). Annual incidence of DHF in Thailand 1973–2009 (Figure S2A, bottom) was obtained from the Annual epidemiological surveillance reports published by the Ministry of Public Health epid.moph.go.th. Dengue serotype prevalence for Thailand 1973–1999 (Figure S2B, top) was based on children hospitalized at the Queen Sirikit National Institute of Child Health in Bangkok, as previously published in [32]. Serotype prevalence in Vietnam (Figure S2B, bottom) is based on data from the southern 20 provinces of Viet Nam over the period 1996–2008, as previously published in [64].

#### Spatial

Spatial serotype data for Ho Chi Minh City (Figure 3B, left panel) was provided by C. Simmons from the Oxford University Clinical Research Unit (OUCRU), Viet Nam, and represents 290 dengue cases in children presenting to the outpatients department of three large hospitals in Ho Chi Minh City during the season 2010—2011. Spatial, or district, information was based on residency of the patient, and the distinction between urban and suburban setting was based on the district's population density according to official census data. Spatial serotype data for Thailand during the 2005/2006 season (Figure 3B, right panel), including spatial information, was obtained from [28], [65].

## Supporting Information

Figure S1 **Epidemic periodicities of a 4-strain pathogen system.** The global wavelet spectrum (GWS) is obtained by averaging the local wavelet power spectrum (LWPS) across time and is analogous to a traditional Fourier spectrum. (***A***) For a directly transmitted pathogen and homogeneous mixing the model generates main epidemic periods that are essentially within the same range for both the pathogen and each of its variants. (***B***) Using a dengue-like framework including a vector population and intrinsic and extrinsic incubation periods increases both the epidemic and serotype-specific periods, which also start to diverge. (***C***) Assuming a spatially structured host and vector population plus seasonality results in a variety of epidemic frequencies for both the pathogen and individual variants. For the pathogen, the strongest period is determined by the annual variation in vector densities, whereas the variants settle close to a 8–9 year periods, similar to those suggested from dengue-endemic regions.
(PDF)

Figure S2 **Characteristic dengue epidemiologies from different endemic settings.** (***A***) Time courses of dengue incidence display strong seasonal signatures (*top*, monthly cases DF/DHF in Mexico) and multi-annual cycles in epidemic outbreaks (*bottom*, annual cases of DHF in Thailand). (***B***) Relative serotype prevalence in Thailand (*top*) and Vietnam (*bottom*) shows sequential replacement in serotype dominance. See Materials and Methods for sources of data.
(PDF)

Figure S3 **Wavelet coherence between communities with increasing spatial distance.** (***A***–***D***)Analysed are the prevalence time series of DENV1 in different communities over a 100 year time course with increased spatial distance along the lattice diagonal, relative to the reference community 1 (corner). The 2D (wavelet coherence) plots show a significant reduction in synchrony between the time series in a time- frequency plane, indicating the loss of similarities (coherence) in serotype behaviour among distant communities. The arrows represent the relative phase, which is a local measure of the delay between the two time series, as a function of scale (frequency) and position (time). Only for communities at close range (*A*, *B*), phase-synchrony can be observed, although peak abundances can remain chaotic and variably uncorrelated due to local demographic stochasticity. Parameters as in Figure 2 of the main text; wavelet coherence was obtained using a Morlet wavelet with a 100 months smoothing window.
(PDF)

Figure S4
**Model sensitivity to changes in parameters relating to** 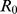 **.** Various human- and vector-associated parameters (rows) were varied and their impact on key epidemiological variables (columns) quantified. (***A***) Transmissibility to humans and mosquitoes with 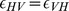; (***B***) Transmissibility to mosquitoes (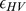) only; (***C***) Transmissibility to humans (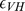) only; (***D***) Intrinsic incubation period (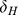); (***E***) extrinsic incubation period (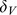); (***F***) number of mosquitoes per human host (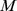). The according basic reproductive number, 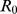, is given by the second x-axis (bottom). The oscillatory behavior in serotype prevalence is maintained given the parameter variations, since the range in serotype epidemic periodicity remains several times above the 1 year (seasonally driven) pathogen epidemic period. The extinction risk is defined as the percent of time individual serotypes remain bellow a critical threshold of 10 infected hosts (human or mosquito), and serotype co-circulation is defined as the percent time where multiple serotypes are present in a given patch (meta-population average). For ease of comparison, epidemiological variables (except age) are normalised to the case of lowest parameter value, with ratios above 1 representing an increase and below 1 a decrease. Dashed lines mark the parameter set of Figure 2 in the main text. Shown are the means and deviations for 25 stochastic simulations. Other parameter values as in Table 1 in the main text.(PDF)

Table S1

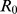
 **estimates for dengue (reproduced from** [**73**] **).**
(PDF)
