# Supplementary material for: Natural, Persistent Oscillations in a Spatial Multi-Strain Disease System with Application to Dengue
Source: PLoS Comput Biol. 2013 Oct 24;9(10):e1003308. doi: 10.1371/journal.pcbi.1003308 (PMC3812071; doi:10.1371/journal.pcbi.1003308)
Supplement: Figure S1 — Epidemic periodicities of a 4-strain pathogen system. The global wavelet spectrum (GWS) is obtained by averaging the local wavelet power spectrum (LWPS) across time and is analogous to a traditional Fourier spectrum. (A) For a directly transmitted pathogen and homogeneous mixing the model generates main epidemic periods that are essentially within the same range for both the pathogen and each of its variants. (B) Using a dengue-like framework including a vector population and intrinsic and extrinsic incubation periods increases both the epidemic and serotype-specific periods, which also start to diverge. (C) Assuming a spatially structured host and vector population plus seasonality results in a variety of epidemic frequencies for both the pathogen and individual variants. For the pathogen, the strongest period is determined by the annual variation in vector densities, whereas the variants settle close to a 8–9 year periods, similar to those suggested from dengue-endemic regions. (PDF) [file pcbi.1003308.s001.pdf]

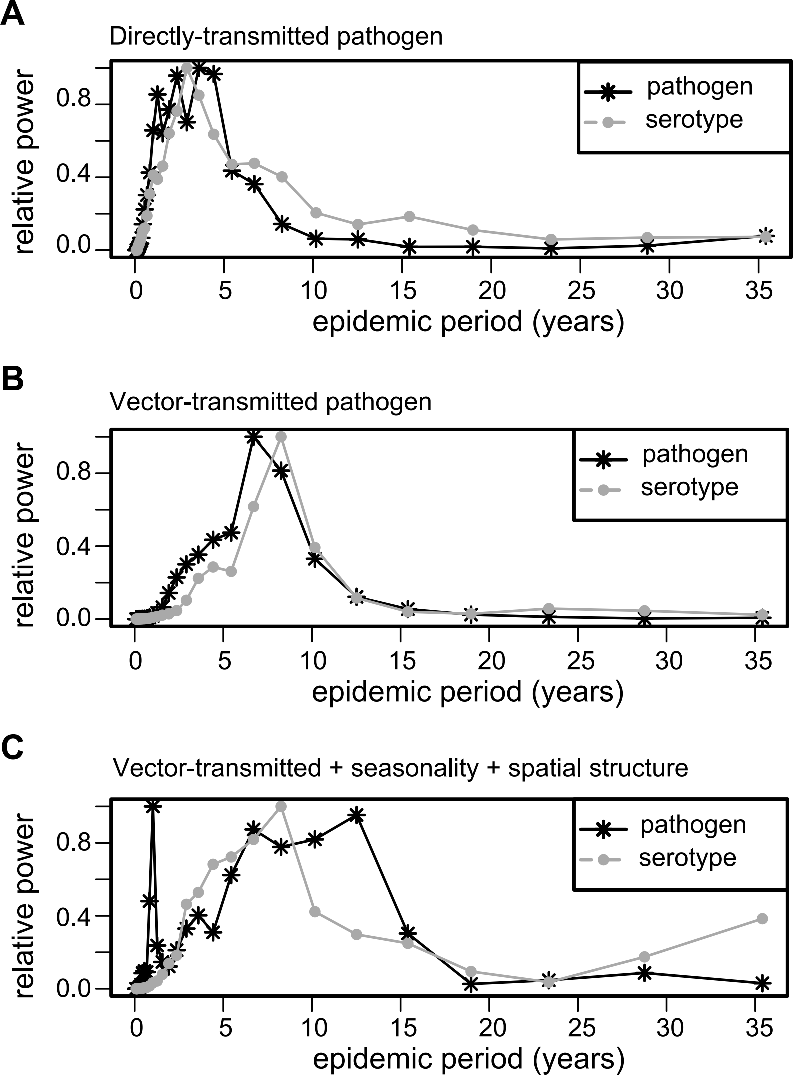

A
Directly-transmitted pathogen
pathogen
serotype
relative power
0.0
0.4
0.8
0
5
10
15
20
25
30
35
epidemic period (years)
B
Vector-transmitted pathogen
pathogen
serotype
relative power
0.0
0.4
0.8
0
5
10
15
20
25
30
35
epidemic period (years)
C
Vector-transmitted + seasonality + spatial structure
pathogen
serotype
relative power
0.0
0.4
0.8
0
5
10
15
20
25
30
35
epidemic period (years)
