# Supplementary material for: Natural, Persistent Oscillations in a Spatial Multi-Strain Disease System with Application to Dengue
Source: PLoS Comput Biol. 2013 Oct 24;9(10):e1003308. doi: 10.1371/journal.pcbi.1003308 (PMC3812071; doi:10.1371/journal.pcbi.1003308)
Supplement: Figure S2 — Characteristic dengue epidemiologies from different endemic settings. (A) Time courses of dengue incidence display strong seasonal signatures (top, monthly cases DF/DHF in Mexico) and multi-annual cycles in epidemic outbreaks (bottom, annual cases of DHF in Thailand). (B) Relative serotype prevalence in Thailand (top) and Vietnam (bottom) shows sequential replacement in serotype dominance. See Materials and Methods for sources of data. (PDF) [file pcbi.1003308.s002.pdf]

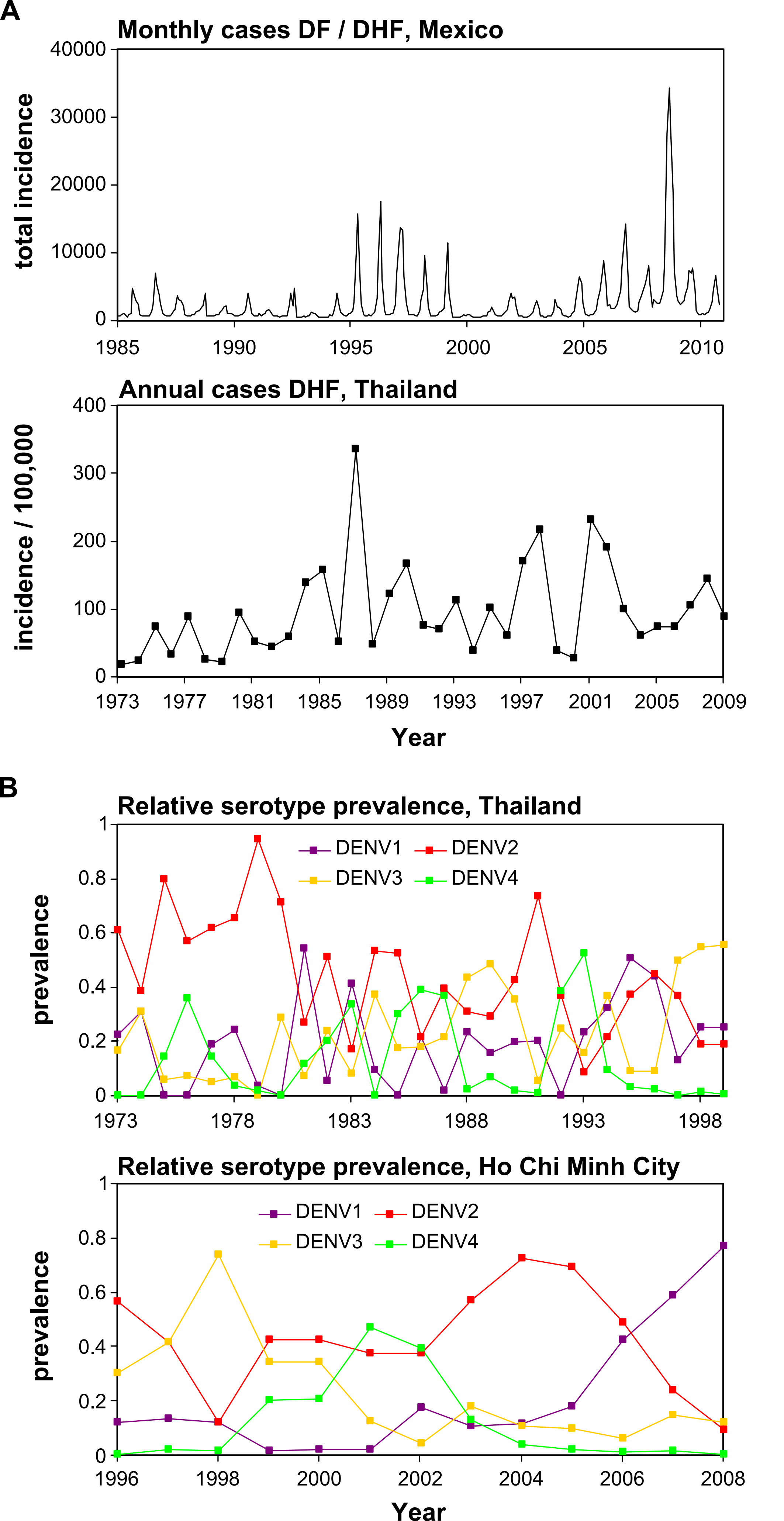
A
Monthly cases DF / DHF, Mexico
total incidence
40000
30000
20000
10000
0
1985
1990
1995
2000
2005
2010
Annual cases DHF, Thailand
incidence / 100,000
400
300
200
100
0
1973
1977
1981
1985
1989
1993
1997
2001
2005
2009
Year
B
Relative serotype prevalence, Thailand
prevalence
1
0.8
0.6
0.4
0.2
0
DENV1
DENV2
DENV3
DENV4
1973
1978
1983
1988
1993
1998
Relative serotype prevalence, Ho Chi Minh City
prevalence
1
0.8
0.6
0.4
0.2
0
DENV1
DENV2
DENV3
DENV4
1996
1998
2000
2002
2004
2006
2008
Year
