# Supplementary figures and images for: Natural, Persistent Oscillations in a Spatial Multi-Strain Disease System with Application to Dengue
Source: PLoS Comput Biol. 2013 Oct 24;9(10):e1003308. doi: 10.1371/journal.pcbi.1003308 (PMC3812071; doi:10.1371/journal.pcbi.1003308)

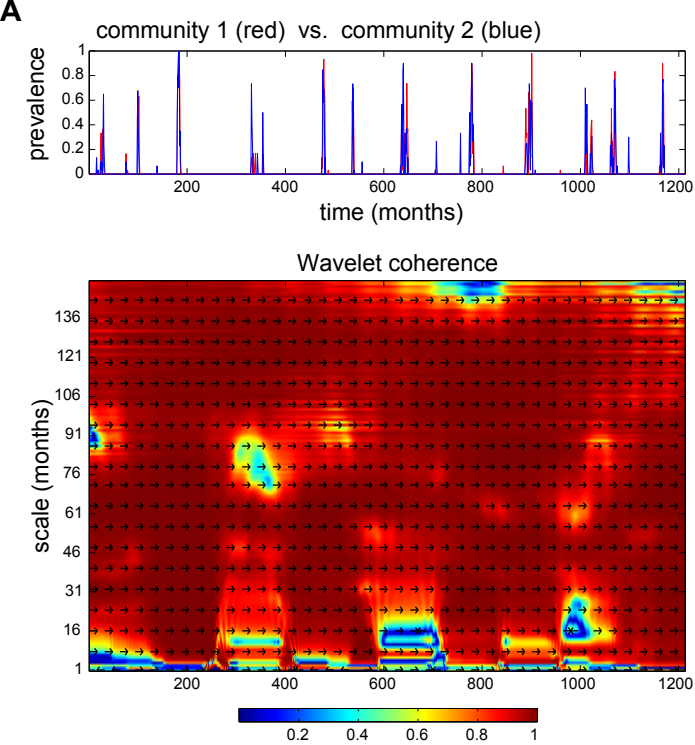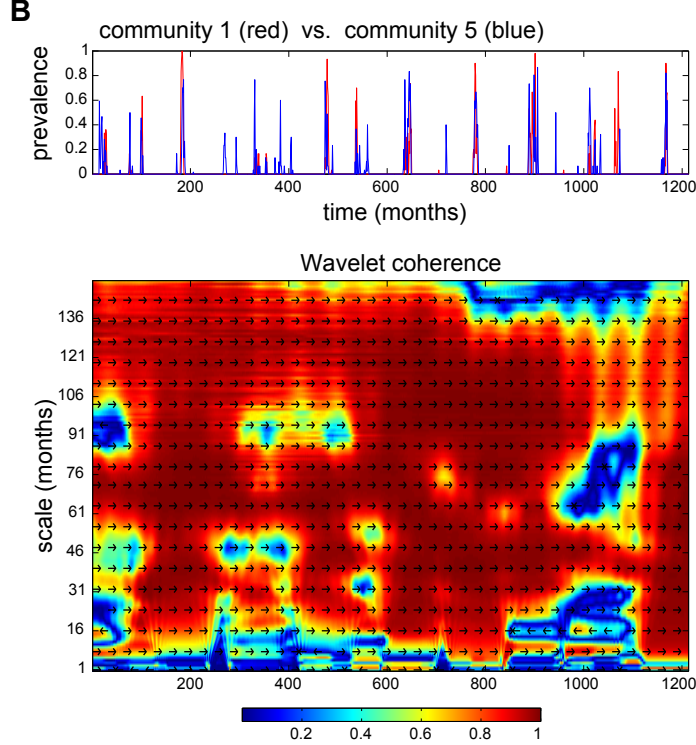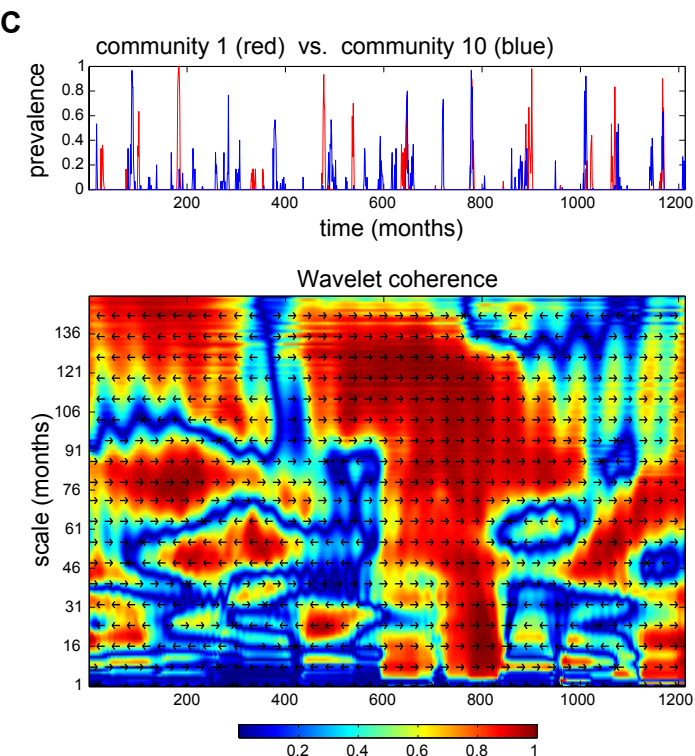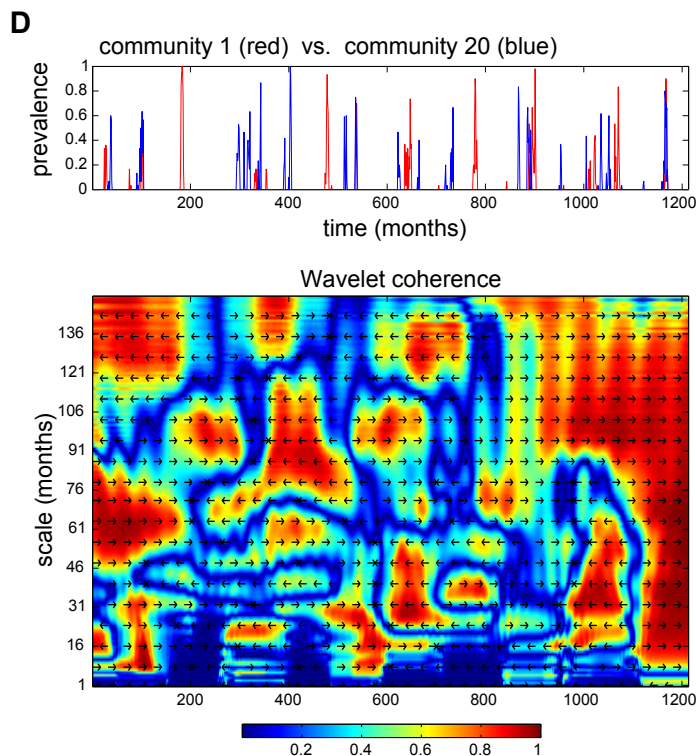

Supplement: Figure S3 — Wavelet coherence between communities with increasing spatial distance. (A–D)Analysed are the prevalence time series of DENV1 in different communities over a 100 year time course with increased spatial distance along the lattice diagonal, relative to the reference community 1 (corner). The 2D (wavelet coherence) plots show a significant reduction in synchrony between the time series in a time- frequency plane, indicating the loss of similarities (coherence) in serotype behaviour among distant communities. The arrows represent the relative phase, which is a local measure of the delay between the two time series, as a function of scale (frequency) and position (time). Only for communities at close range (A, B), phase-synchrony can be observed, although peak abundances can remain chaotic and variably uncorrelated due to local demographic stochasticity. Parameters as in Figure 2 of the main text; wavelet coherence was obtained using a Morlet wavelet with a 100 months smoothing window. (PDF) [file pcbi.1003308.s003.pdf]

A

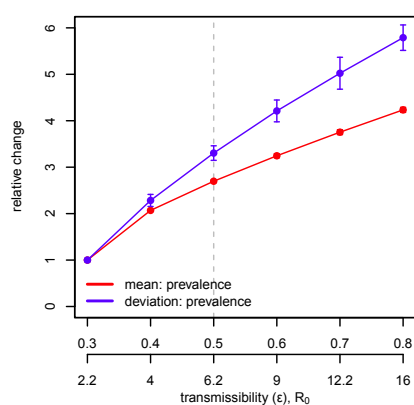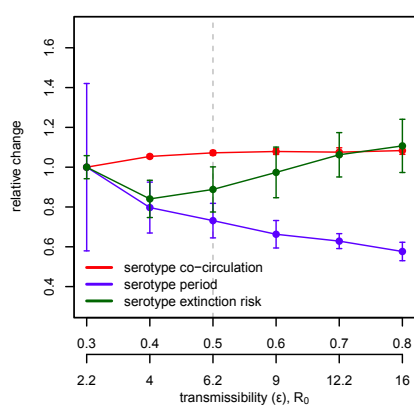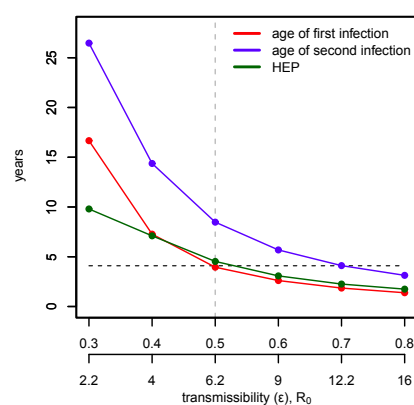

B

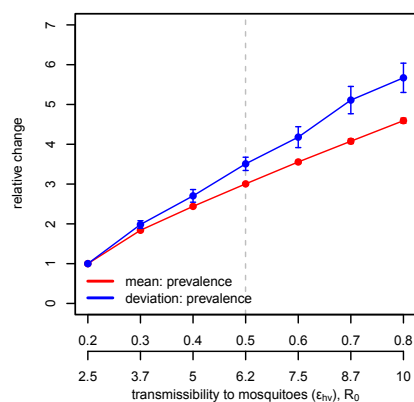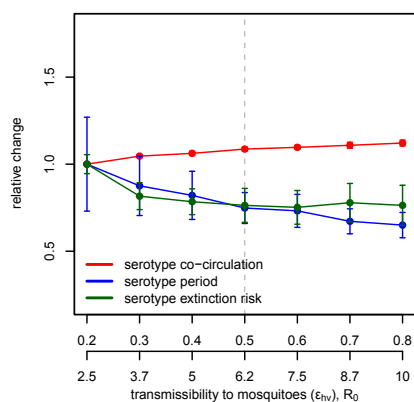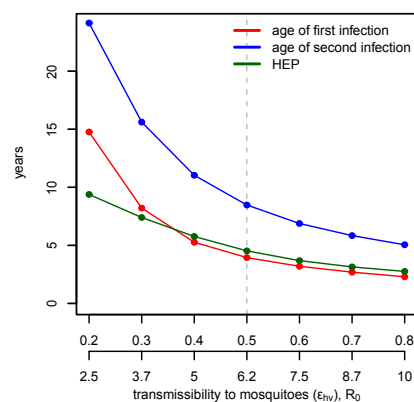

C

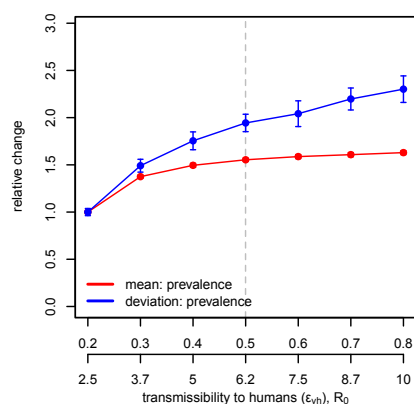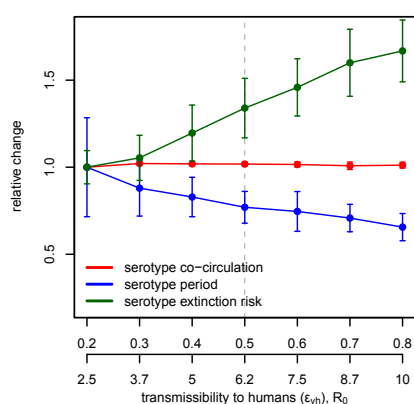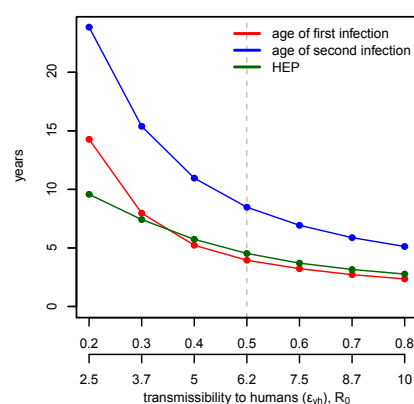

D

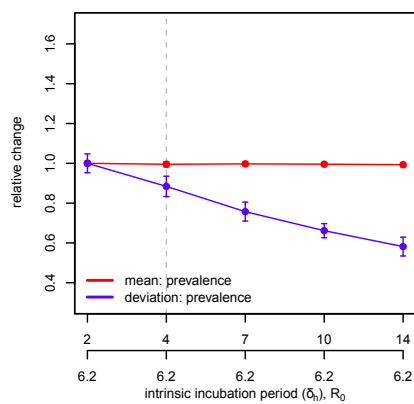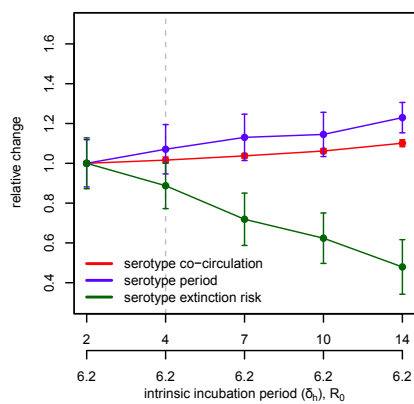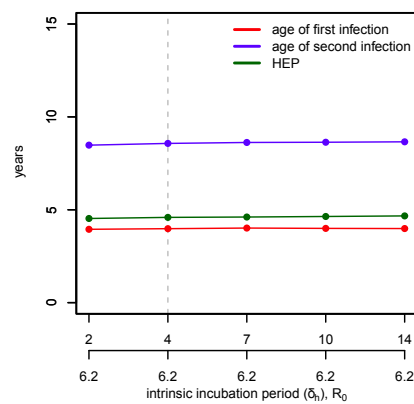

E

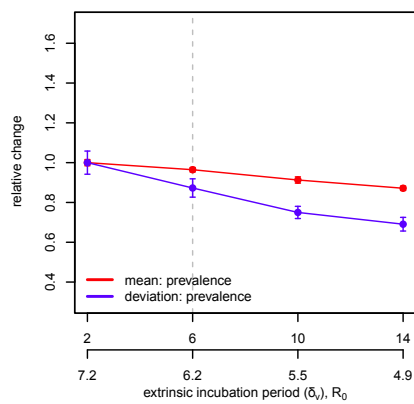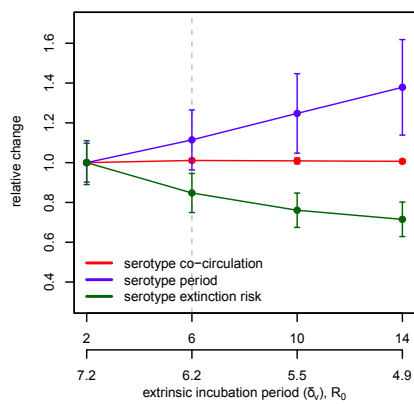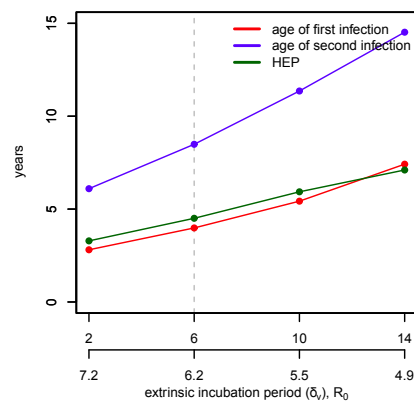

F

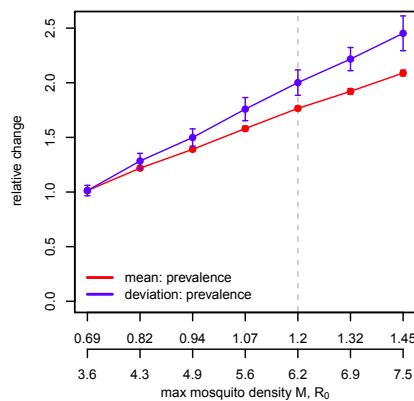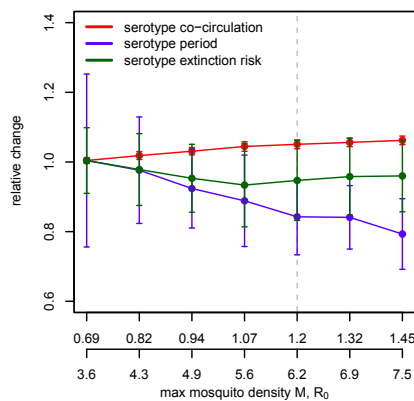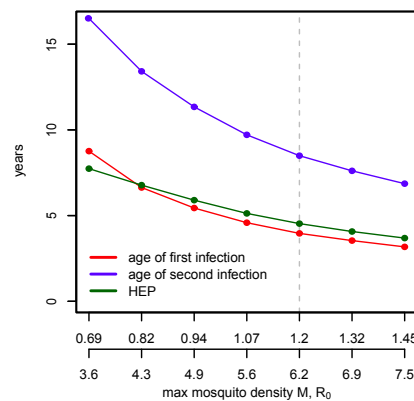

Supplement: Figure S4 — Model sensitivity to changes in parameters relating to . Various human- and vector-associated parameters (rows) were varied and their impact on key epidemiological variables (columns) quantified. (A) Transmissibility to humans and mosquitoes with ; (B) Transmissibility to mosquitoes () only; (C) Transmissibility to humans () only; (D) Intrinsic incubation period (); (E) extrinsic incubation period (); (F) number of mosquitoes per human host (). The according basic reproductive number, , is given by the second x-axis (bottom). The oscillatory behavior in serotype prevalence is maintained given the parameter variations, since the range in serotype epidemic periodicity remains several times above the 1 year (seasonally driven) pathogen epidemic period. The extinction risk is defined as the percent of time individual serotypes remain bellow a critical threshold of 10 infected hosts (human or mosquito), and serotype co-circulation is defined as the percent time where multiple serotypes are present in a given patch (meta-population average). For ease of comparison, epidemiological variables (except age) are normalised to the case of lowest parameter value, with ratios above 1 representing an increase and below 1 a decrease. Dashed lines mark the parameter set of Figure 2 in the main text. Shown are the means and deviations for 25 stochastic simulations. Other parameter values as in Table 1 in the main text. (PDF) [file pcbi.1003308.s004.pdf]
