# Supplementary material for: Natural, Persistent Oscillations in a Spatial Multi-Strain Disease System with Application to Dengue
Source: PLoS Comput Biol. 2013 Oct 24;9(10):e1003308. doi: 10.1371/journal.pcbi.1003308 (PMC3812071; doi:10.1371/journal.pcbi.1003308)
Supplement: Table S1 — estimates for dengue (reproduced from [73] ). (PDF) [file pcbi.1003308.s005.pdf]

Table S1: R_0 **estimates for dengue (reproduced from [13]).**

Study	N	Cross-protection	Enhancement	Median R_0	Method
[1]	1	No	No	1.3 (0, 2.4)	final epi. size
[2]	1	No	No	1.9	hypothetical
[3]	1	No	No	2.0 (1.6, 2.5)	epi. growth curve
[4]	4	Susceptibility	Susceptibility	4.8 (1.4, 8.5)	age stratified seroprev.
[5]	1	No	No	6.3 (3.6,12.9)	epi. growth curve
[6]	1	No	No	4.8 (2.7, 11.6)	epi. growth curve
[7]	1	No	No	3.0 (2.0, 103)	epi. growth curve
[8]	1	No	No	2.0 (0.5, 3.3)	epi. growth curve
[9]	1	No	No	3.69 (2.26, 11.39)	epi. growth curve
[10]	4	Susceptibility	Both*	$ (4, 12)	age dist. of cases
[11]	1	No	No	5.17 (1.56, 22.89)	fitting SIR model
[12]	4	Susceptibility	Susceptibility	(5.2, 6.7)	age dist. of cases

N - number of serotypes
* - susceptibility and infectiousness

$ - only a range was explored.

References:

[**1**] Koopman, J. S., Prevots, D. R., Vaca Marin, M. a., Gomez Dantes, H., Zarate Aquino, M. L., Longini, I. M., and Sepulveda Amor, J. (1991). Determinants and predictors of dengue infection in Mexico. American journal of epidemiology, 133(11):1168–78.

[**2**] Newton, E. a. and Reiter, P. (1992). A model of the transmission of dengue fever with an evaluation of the impact of ultra-low volume (ULV) insecticide applications on dengue epidemics. The American journal of tropical medicine and hygiene, 47(6):709–20.

[**3**] Marques, C. A., Forattini, O. P., and Massad, E. (1994). The basic reproductionnumber for dengue fever in Sao Paulo state, Brazil: 1990-1991 epidemic. Transactions of the Royal Society of Tropical Medicine and Hygiene, 88(1):58–9.

[**4**] Ferguson, N. M., Donnelly, C. A., and Anderson, R. M. (1999b). Transmission dynamics and epidemiology of dengue: insights from age-stratified sero-prevalence surveys. Philosophical transactions of the Royal Society of London. Series B, Biological sciences, 354(1384):757–68.

[**5**] Massad, E., Coutinho, F. A., Burattini, M. N., and Lopez, L. F. (2001). The risk of yellow fever in a dengue-infested area. Transactions of the Royal Society of Tropical Medicine and Hygiene, 95(4):370–4.

[**6**] Massad, E., Burattini, M. N., Coutinho, F. A. B., and Lopez, L. F. (2003). Dengue and the risk of urban yellow fever reintroduction in Sao Paulo State, Brazil. Revista de saude publica, 37(4):477–84.

[**7**] Favier, C., Degallier, N., Rosa-Freitas, M. G., Boulanger, J. P., Costa Lima, J. R., Luitgards-Moura, J. F., Menkes, C. E., Mondet, B., Oliveira, C., Weimann, E. T. S., and Tsouris, P. (2006). Early determination of the reproductive number for vector-borne diseases: the case of dengue in Brazil. Tropical medicine & international health : TM & IH, 11(3):332–40.

[**8**] Chowell, G., Diaz-Duenas, P., Miller, J. C., Alcazar-Velazco, A., Hyman, J. M., Fenimore, P. W., and Castillo-Chavez, C. (2007). Estimation of the reproduction number of dengue fever

from spatial epidemic data. Mathematical biosciences, 208(2):571–89.

[**9**] Coelho, G. E., Burattini, M. N., Teixeira, M. D. G., Coutinho, F. A. B., and Massad, E. (2008). Dynamics of the 2006/2007 dengue outbreak in Brazil. Memorias do Instituto Oswaldo Cruz, 103(6):535–9.

[**10**] Nagao, Y. and Koelle, K. (2008). Decreases in dengue transmission may act to increase the incidence of dengue hemorrhagic fever. Proceedings of the National Academy of Sciences of the United States of America, 105(6):2238–43.

[**11**] Degallier, N., Favier, C., Boulanger, J.-P., and Menkes, C. (2009). Imported and autochthonous cases in the dynamics of dengue epidemics in Brazil. Revista de saude publica, 43(1):1–7.

[**12**] Cummings, D. a. T., Iamsirithaworn, S., Lessler, J. T., McDermott, A., Prasanthong, R., Nisalak, A., Jarman, R. G., Burke, D. S., and Gibbons, R. V. (2009). The impact of the demographic transition on dengue in Thailand: insights from a statistical analysis and mathematical modeling. PLoS Medicine, 6(9):e1000139.

[**13**] Johansson, M. a., Hombach, J., and Cummings, D. a. T. (2011). Models of the impact of dengue vaccines: A review of current research and potential approaches. Vaccine, pages 1–9.
